# Echinacoside inhibited cardiomyocyte pyroptosis and improved heart function of HF rats induced by isoproterenol via suppressing NADPH/ROS/ER stress

**DOI:** 10.1111/jcmm.17564

**Published:** 2022-10-06

**Authors:** YaJuan Ni, Jing Zhang, Wenjing Zhu, Yixuan Duan, HongYuan Bai, Chunhong Luan

**Affiliations:** ^1^ Department of Cardiology The Second Affiliated Hospital of Xi'an Jiaotong University Xi'an China; ^2^ Department of Cardiology The First Affiliated Hospital of Xi'an Jiaotong University Xi'an China

**Keywords:** echinacoside, endoplasmic reticulum stress, heart failure, heart function, pyroptosis

## Abstract

Prevalence of heart failure (HF) continues to rise over time and is a global difficult problem; new drug targets are urgently needed. In recent years, pyroptosis is confirmed to promote cardiac remodelling and HF. Echinacoside (ECH) is a natural phenylethanoid glycoside and is the major active component of traditional Chinese medicine Cistanches Herba, which is reported to possess powerful anti‐oxidation and anti‐inflammatory effects. In addition, we previously reported that ECH reversed cardiac remodelling and improved heart function, but the effect of ECH on pyroptosis has not been studied. So, we investigated the effects of ECH on cardiomyocyte pyroptosis and the underlying mechanisms. In vivo, we established HF rat models induced by isoproterenol (ISO) and pre‐treated with ECH. Indexes of heart function, pyroptotic marker proteins, ROS levels, and the expressions of NOX2, NOX4 and ER stress were measured. In vitro, primary cardiomyocytes of neonatal rats were treated with ISO and ECH; ASC speckles and caspase‐1 mediated pyroptosis in cardiomyocytes were detected. Hoechst/PI staining was also used to evaluate pyroptosis. ROS levels, pyroptotic marker proteins, NOX2, NOX4 and ER stress levels were all tested. In vivo, we found that ECH effectively inhibited pyroptosis, down‐regulated NOX2 and NOX4, decreased ROS levels, suppressed ER stress and improved heart function. In vitro, ECH reduced cardiomyocyte pyroptosis and suppressed NADPH/ROS/ER stress. We concluded that ECH inhibited cardiomyocyte pyroptosis and improved heart function via suppressing NADPH/ROS/ER stress.

## 1 INTRODUCTION

Heart failure (HF) is a clinical syndrome caused by the structural or functional impairment of ventricular filling or the ejection of blood, the prevalence continues to rise over time, which is a global difficult problem. Projections show that the prevalence of HF will increase 46% from 2012 to 2030, resulting in >8 million people ≥18 years of age with HF in the United States.^1^ During the past 20 years, drug treatments for HF have made great progress, but the effect is very limited, so new drugs are urgently needed.

Pyroptosis is a necrotic form of regulated cell death characterized by plasma membrane permeabilization, rupture and DNA damage.^2^, ^3^ It is a proinflammatory and new‐found type of programmed cell death, because pyroptotic cells release proinflammatory intracellular molecules, including IL‐1 family cytokines and damage‐associated molecular patterns (DAMPs), and caspases that induce pyroptosis (caspase‐1/4/5/11) are called inflammatory caspases. In contrast to pyroptosis, apoptosis is characterized by cell shrinkage, membrane blebbing, phosphatidylserine (PS) externalization, nuclear DNA fragmentation and nuclear condensation. In addition, apoptosis is mainly induced by caspase‐8 and caspase‐9, which activate downstream effector caspases, such as caspase‐3 and caspase‐7, and then, a number of substrates, including Rho‐associated protein kinase I, ATPase 11A/C, Xk‐related protein 8, and inhibitor of caspase‐activated DNase are cleaved. The cleavage of so‐called death substrates leads to apoptosis.^3^, ^4^

NOD‐like receptor protein 3 (NLRP3) inflammasome is a key factor of pyroptosis, and after activation, NLRP3 interacts with apoptosis‐associated speck‐like protein (ASC), recruits and cleaves pro‐caspase‐1, thus activating caspase‐1, which cleaves gasdermin D(GSDMD) with producing GSDMD‐N, and further triggers the pyroptotic cascade (activated caspase‐1 induces inflammation by cleaving pre‐interleukin [IL]‐1β and pre‐IL‐18 into IL‐1β and IL‐18, which are biomarkers of pyroptotic).^5^ In recent years, studies confirmed that NLRP3 inflammasome is activated during several cardiac disorders, and NLRP3 inflammasome‐mediated pyroptosis aggravates pressure overload‐induced cardiac hypertrophy, fibrosis, dysfunction,^6^ which is a key pathological factor underlying HF.^7^, ^8^, ^9^

ROS acts as a major trigger of NLRP3 inflammasome activation,^10^ NADPH oxidase, mainly subunit NOX2 and NOX4, is the main source of intracellular ROS, and the expression of NADPH is up‐regulated in HF.^11^, ^12^ ER is essential for protein synthesis, protein folding, protein translocation and calcium homoeostasis.^13^ Changes in the cellular oxidation stress generation can compromise ER homeostasis, leading to an accumulation of misfolded and unfolded proteins, which activates unfolded protein response (UPR).^14^ In HF, the upregulation of NOX2 and NOX4 leads to an increase in generation of ROS and ER stress via NOX4/ROS pathway, vice versa, ER stress and UPR cause a further increase of intracellular ROS.^15^ So, it seems to be a vicious circle, and it leads ROS excessive release.

Echinacoside (ECH) is a natural phenylethanoid glycoside and is the major active component of traditional Chinese medicine Cistanches Herba. In recent years, ECH has been reported to possess a variety of pharmacological effects, such as antioxidant, anti‐inflammatory, anti‐apoptosis and anti‐tumour properties.^16^, ^17^ Our previous study indicated that ECH reversed cardiac remodelling and improved heart function via inhibiting mitochondria ROS.^18^ However, pyroptosis is an essential factor of cardiac remodelling and HF, but it remains unknown whether ECH can suppress cardiomyocyte pyroptosis via inhibiting NADPH/ROS/ER stress. In present study, in vivo, we confirmed that ECH suppressed pyroptosis and improved heart function via down‐regulating NADPH/ROS/ER stress. In vitro, we also demonstrated that ECH suppressed cardiomyocyte pyroptosis via down‐regulating NADPH/ROS and ER stress. The results are intending to provide experimental evidence for the development of new drugs to prevent HF.

## 2 MATERIALS AND METHODS

### 2.1 Animals and treatment

Five‐week‐old Sprague–Dawley male rats weighing 120–140 g were acquired from Xi'an Jiaotong University Laboratorial Animal Center (Shaanxi, China). The investigation conformed to The Guide for the Care and Use of Laboratory Animals, published by the US National Institutes of Health (NIH publication no. 85‐23, revised in 1996). Rat model of HF was induced by intraperitoneal injection of ISO (10 mg/kg; Sigma) as described previously by us,^18^, ^19^ in ECH group, ECH (20 μg/g; MCE) was administered by the same way at 30 min before ISO was treated. Control animals were administrated with 0.9% NaCl. The treatments were administrated once daily and lasted for 2 weeks. There were six animals in each group, after 2 weeks of treatment, echocardiographic measurements were performed.

### 2.2 Echocardiographic measurements

Anaesthesia was induced by intraperitoneal injection of chloral hydrate (300 mg/kg). SONOS‐2500 ultrasound system with an ultrasound transducer of 7.5 MHz (HP) was used to detect heart structure and function. The left ventricular ejection fraction (LVEF), left ventricular fractional shortening (LVFS), left ventricular end‐diastolic dimensions (LVIDd), left ventricular end‐systolic dimensions (LVIDs) and heart rate (HR) were measured.

### 2.3 Immunohistochemical staining (IHC)

Left ventricular myocardial tissue was fixed, paraffin embedded and sectioned, incubated at room temperature with 3% H_2_O_2_ for 5–10 min, washed with distilled water and immersed into PBS for 5 min, twice. Incubated 10% goat serum at room temperature for 10 min, and then absorbed the serum. Primary antibodies of NOX2, NOX4, GRP78 and CHOP were added and incubated at 37°C for 2 h. Washed with PBS, three times for 5 min. Added secondary antibodies and incubated at 37°C for 30 min. Washed with PBS, three times for 5 min. Alkaline phosphatase‐labelled streptomycin working solution was added and incubated at 37°C for 30 min. Washed with PBS, DAB reagent was added and incubated at 37°C for 10 min, washed and re‐dyed, dehydrated and sealed with neutral balsam.

### 2.4 ROS in myocardial tissue was detected with DCFH‐DA fluorescent probes

Fresh left ventricular myocardial tissue homogenate was fabricated by homogenizer; BCA protein assay kit (Bio tech) was used to determine the concentration of the protein. DCFH‐DA detection kit was used to measure the level of ROS following the supplier's instructions. Fluorescence intensity was measured with an enzyme‐labelling measuring instrument; results were represented as RLU/mg protein.

### 2.5 Cell culture and treatment

The primary cardiomyocytes of neonatal rats were isolated from Newborn SD rats within 3 days. The rats were acquired from Xi'an Jiao tong University Laboratorial Animal Center (Shaanxi, China). The ventricular parts were placed in a petri dish containing 1× ADS (10× ADS formula: NaCl 68 g, HEPES 47.6 g, Na 2 HPO 4 1.38 g, Glucose 6 g, KCl 4 g, MgSO_4_·7H_2_O 0.51 g). Tissue was cut into pieces until homogenized and added trypsin collagenase digestion solution, and beat evenly and put into 37°C shaker for 10 min, the digestion was terminated with horse serum, centrifugated at 1000 rpm for 5 min, resuspended with cell culture medium (DMEM high glucose medium + 10% horse serum + 5% FBS + 1% penicillomycin) and sticked to the wall for 30 min, centrifuged at 1000 r/min for 5 min and resuspended with 1× ADS. Density gradient centrifugation was used to separate fibroblast suspension (upper layer) and cardiomyocyte cell suspension(lower layer), culture medium was added and centrifuged at 1000 rpm for 5 min, and suspension was performed with cardiomyocyte culture. Neonatal rat cardiomyocytes were obtained and placed on corresponding culture plates.

### 2.6 Caspace‐1 was detected by flow cytometry

Cells at logarithmic growth stage were taken; ISO (Sigma) group cells were incubated with medium containing 10 μM ISO (Sigma) for 24 h; ECH group cells were pre‐treated with 50 μM ECH for 30 min prior ISO exposure treated with ISO; control cells (Ctrl) were treated with PBS and without any drugs. Cells were collected and centrifuged for 5 min, 1200 r/min, resuspended with 300 μl PBS, then added 30× FLICA solution and incubated at 37°C for 1 h away from light (Ctrl cells were not added FLICA and PI; Positive control 1, the solvent group with the most obvious apoptotic effect was taken as positive control, and only FLICA single label was added; Positive control 2, the solvent group with the most obvious apoptotic effect was used as positive control, and only PI single standard was added). 1× Apoptosis Wash Buffer was added, centrifuged at 1200 r/min for 5 min, washed with 1× Apoptosis Wash Buffer and centrifuged again, then resuspended in 1× Apoptosis Wash Buffer and added PI, the cells were detected by flow cytometry.

### 2.7 Intracellular ROS was measured by flow cytometry

After treatment, cells were trypsinized and washed with PBS, resuspended in PBS, stained with DCFH‐DA (Beyotime) at the final concentration of 1 μM and incubated for 1 h at 37°C in an incubator with CO_2_ and then tested in a flow cytometer at 488 nm excitation. The fluorescence intensity represents the level of intracellular ROS.

### 2.8 ASC speckles were detected by immunofluorescence staining (IFC)

The slides of cells were washed by PBS, fixed with 4% paraformaldehyde for 15 min, washed three times and treated with 0.5%Triton X‐100 for 20 min, washed three times and blocked with normal goat serum, then incubated with primary ASC antibody (1:200 dilution; Santa Cruz Biotechnology). Then, incubated with FITC‐labelled fluorescent secondary antibody. DAPI was added to hide from light and incubated for 5 min washed four times with PBST; the slides were sealed with a sealing solution containing an anti‐fluorescence quenching agent. The cells were observed under a fluorescence microscope.

### 2.9 Pyroptosis was detected by Hoechst/PI staining

Cells at logarithmic growth stage were taken, ISO and ECH were treated as mentioned above, discarded the medium in the six‐well plate, and added 1 ml cell staining buffer, 5 μl Hoechst staining fluid and 5 μl PI staining fluid (Beijing Solebo Technology Co., LTD.) to each well, after mixing, incubated for 20 min in the refrigerator at 4°C, washed with PBS, the cells were observed under a fluorescence microscope.

### 2.10 Western blot

Total protein was extracted from left ventricular of rats and primary cardiomyocytes of neonatal rats (newborn SD rats within 3 days); Western blot was performed according to the procedures described by us previously^20^; protein concentration was determined by BCA protein assay kit (B&C Biotech); antibodies of NOX2, NOX4, IRE1α, p‐IRE1α, PERK, p‐PERK, ATF6, CHOP, caspase‐1, cleaved caspase‐1, NLRP3, IL‐1β, IL‐18, GSDMD‐N, p‐SAPK/JNK and ASC were diluted to 1:200 (Santa Cruz). Protein bands were detected by a chemiluminescence system (ChemiDoc XRS; BioRad), Mean value INT(Intensity) of every band was measured, and the relative levels of proteins were calculated by normalizing the value to GAPDH, and the ratios of cleaved caspase‐1/ caspase‐1, p‐IRE1α/ IRE1α and p‐PERK/PERK were calculated.

### 2.11 Statistical analysis

Data were presented as means ± *SEM*. All statistics were determined using SPSS15.0 software (SPSS Inc.). Comparisons between two groups were performed with Student's *t*‐test and among three groups were compared with one‐way anova followed by Tukey post hoc test for significance. A probability value of *p* < 0.05 is considered significant.

## 3 RESULTS

### 3.1 ECH effectually suppressed cardiomyocyte pyroptosis and protected against HF of rats induced by ISO

**FIGURE 1:**
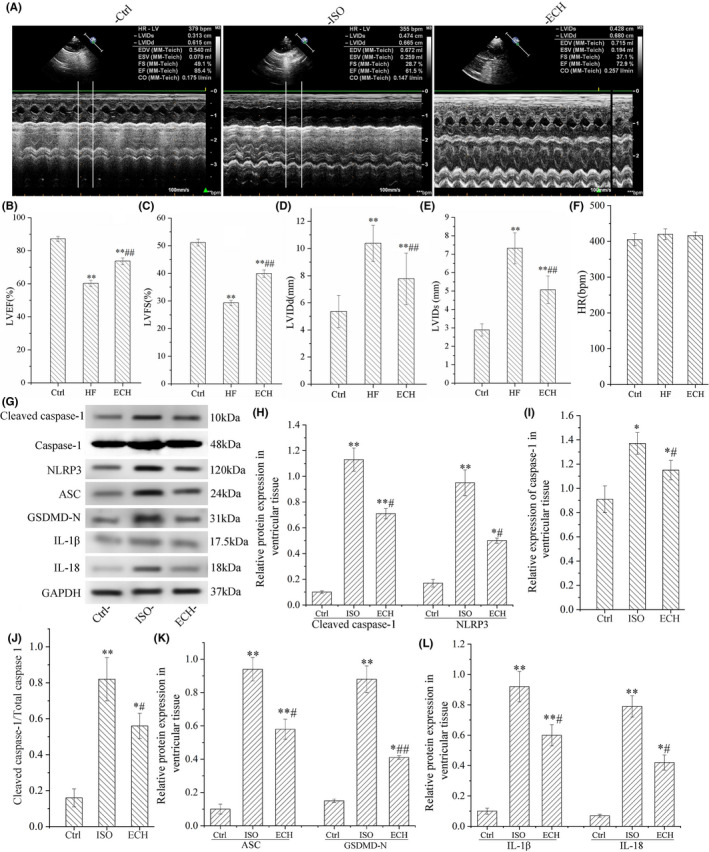
ECH effectively inhibited cardiomyocyte pyroptosis and improved heart function of HF rats induced by ISO. (A) Representative echocardiographic image of Ctrl (*n* = 6), ISO (*n* = 6) and ECH (*n* = 6) treated rat. (B–E) ECH effectively decreased LVIDd, LVIDs and increased LVEF, LVFS. (F) There is no significant difference in HR among three groups. (G) Representative bands of Western blot of cleaved caspase‐1, caspase‐1, NLRP3, ASC, GSDMD‐N, IL‐1β and IL‐18. (H–L) Statistical histogram of relative protein expression/GAPDH and the ratio of cleaved caspase‐1/caspase‐1. All *p < 0.05 versus Ctrl, **p < 0.01 versus Ctrl; #*p* < 0.05 versus ISO, ##*p* < 0.01 versus ISO. Error bars represent *SD*.

Echocardiography results showed that LVFS and LVEF were significantly reduced and that LVIDd and LVIDs were significantly increased in ISO group. However, ECH significantly decreased LVIDd and LVIDs, and effectively improved LVEF and LVFS, there was no significant difference in HR between the three groups, as shown in Figure 1A–F. The protein markers of pyroptosis in left ventricular tissue of rats were tested by WB, the results revealed that the expression of cleaved caspase‐1, caspase‐1, the ratio of cleaved caspase‐1/caspase‐1, NLRP3, IL‐1β, IL‐18, GSDMD‐N and ASC were significantly up‐regulated in HF rats induced by ISO, and obviously, ECH reversed the changes and down‐regulated the expression of these proteins, as shown in Figure 1G–L. The data indicated that ISO induced pyroptosis of many cardiomyocyte and contributed to HF, but ECH effectually suppressed cardiomyocyte pyroptosis and improved heart function.

### 3.2 ECH significantly down‐regulated NOX2, NOX4 and ROS and inhibited ER stress induced by ISO

**FIGURE 2:**
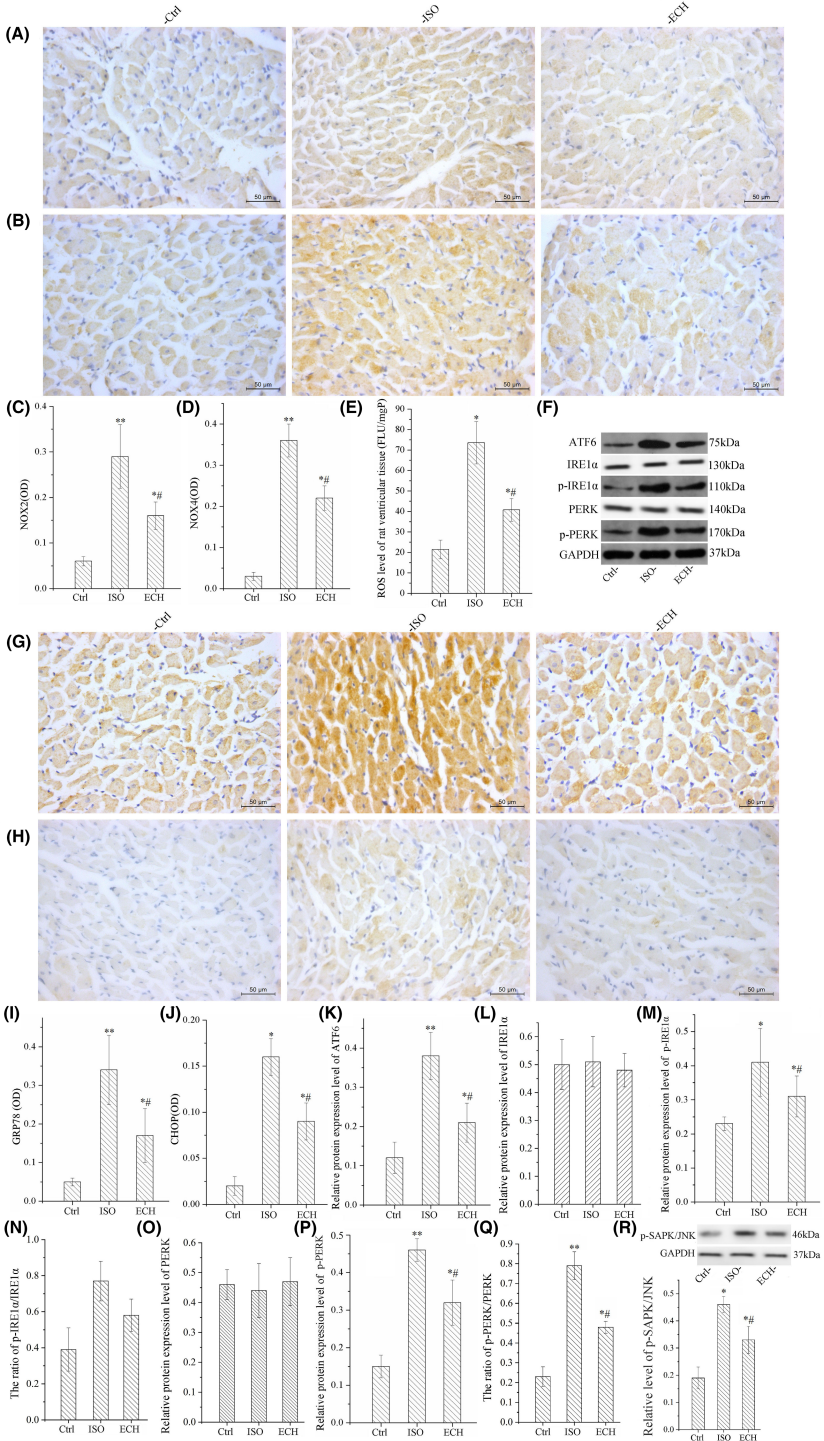
ECH significantly suppressed NOX2, NOX4, p‐SAPK/JNK, ROS and ER stress in left ventricular tissue of HF rats induced by ISO. (A, B) Representative immunohistochemical image of NOX2 and NOX4 in Ctrl, ISO and ECH‐treated rats. (C, D) Statistical histogram of mean optical density (OD) of NOX2 and NOX4 in rats. (E) ROS level in left ventricular tissue of Ctrl, ISO and ECH‐treated rats. (F) Representative bands of Western blot of ATF6, IRE1α, p‐IRE1α, PERK and p‐PERK in Ctrl, ISO and ECH‐treated rats. (G, H) Representative immunohistochemical image of GRP78 and CHOP in Ctrl, ISO and ECH‐treated rats. (I, J) Statistical histogram of OD of GRP78 and CHOP. (K–Q) Statistical histogram of relative ATF6, p‐IRE1α, p‐PERK expression/GAPDH and the ratios of p‐IRE1α/IRE1α, p‐PERK/PERK. (R) Representative bands of Western blot of p‐SAPK/JNK and statistical histogram. All *p < 0.05 versus Ctrl, **p < 0.01 versus Ctrl; #*p* < 0.05 versus ISO, ##*p* < 0.01 versus ISO. Error bars represent *SD*.

NOX2 and NOX4 were the main source of intracellular ROS, their expression was evidently increased in ISO group, and ECH reversed the changes, as shown in Figure 2A–D. As phosphorylation SAPK‐JNK(p‐ SAPK‐JNK) was an activator of NADPH,^21^ we also detected the level of p‐SAPK‐JNK in three groups. Consistent with this, the level of p‐SAPK‐JNK in ISO increased, and ECH decreased it obviously (Figure 2R). The ROS, which induced ER stress and acted as a major trigger of NLRP3 inflammasome activation, was also excessive release in ISO group and was also inhibited by ECH, as shown in Figure 2E. The expression of protein markers of ER stress GRP78, IRE1α, p‐IRE1α, PERK, p‐PERK, ATF6 and CHOP were detected by WB and IHC, the results indicated that the expression of GRP78, p‐IRE1α, p‐PERK, ATF6, CHOP and ratios of p‐IRE1α/IRE1α and p‐PERK/PERK were all remarkably up‐regulated in ISO rats, but ECH treatment distinctly down‐regulated these protein expression and alleviated ER stress, but there were no significant differences in expression of IRE1α and PERK among three groups, as shown in Figure 2F–Q.

### 3.3 ECH evidently inhibited ISO induced cardiomyocyte pyroptosis in vitro

**FIGURE 3:**
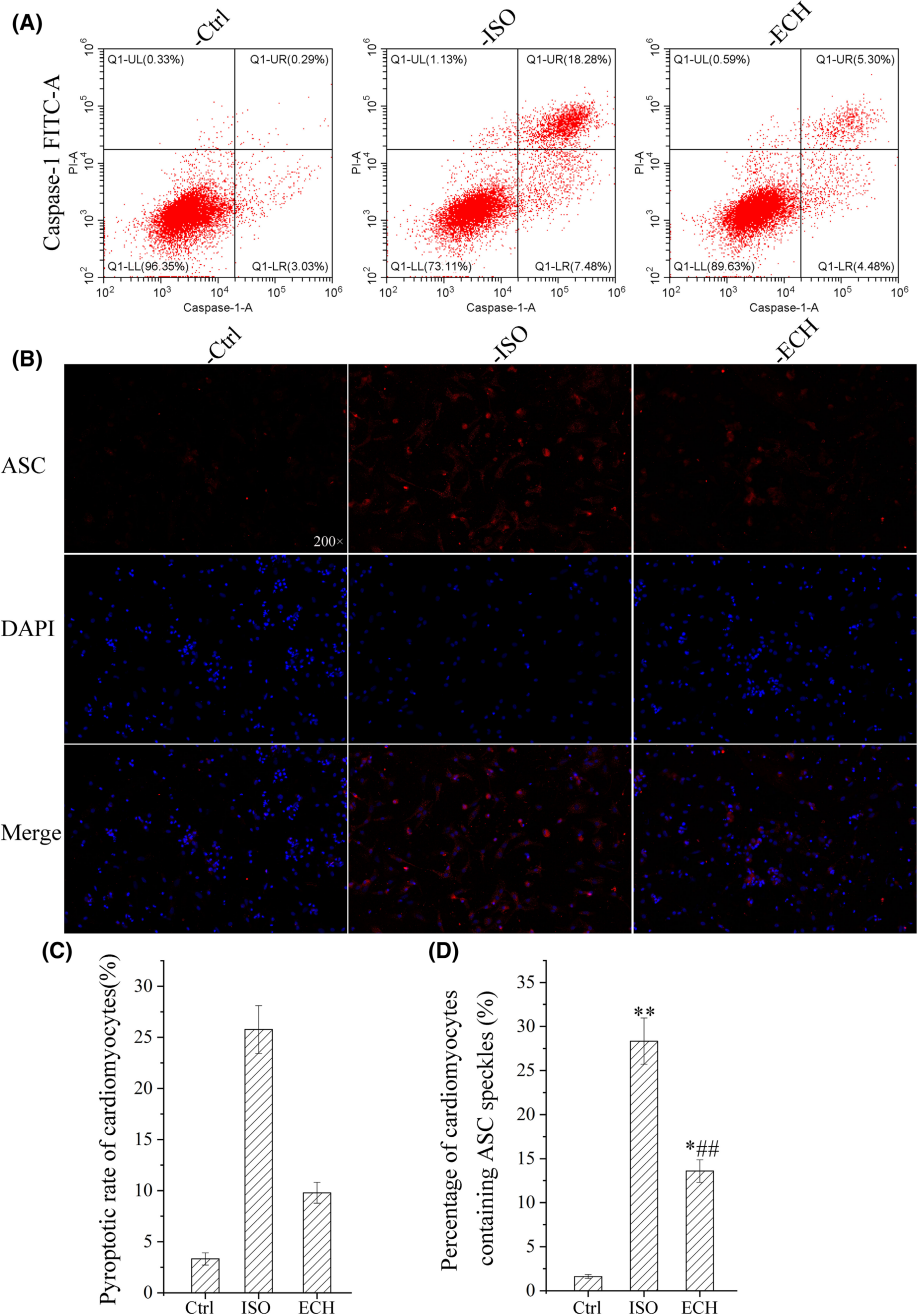
ECH reduced caspase‐1 mediated pyroptosis and ASC speckles in primary cardiomyocytes of neonatal rat induced by ISO. (A) Representative flow cytometry image of caspase‐1 mediated pyroptosis in Ctrl, ISO and ECH‐treated cardiomyocytes. (B) Representative immunofluorescence staining image of ASC in Ctrl, ISO and ECH‐treated cardiomyocytes. (C) Statistical histogram of cardiomyocyte pyroptotic rate mediated by caspase‐1. (D) Statistical histogram of ASC speckles positive rate of cardiomyocytes. All *p < 0.05 versus Ctrl, **p < 0.01 versus Ctrl; #*p* < 0.05 versus ISO, ##*p* < 0.01 versus ISO. Error bars represent *SD*.

**FIGURE 4:**
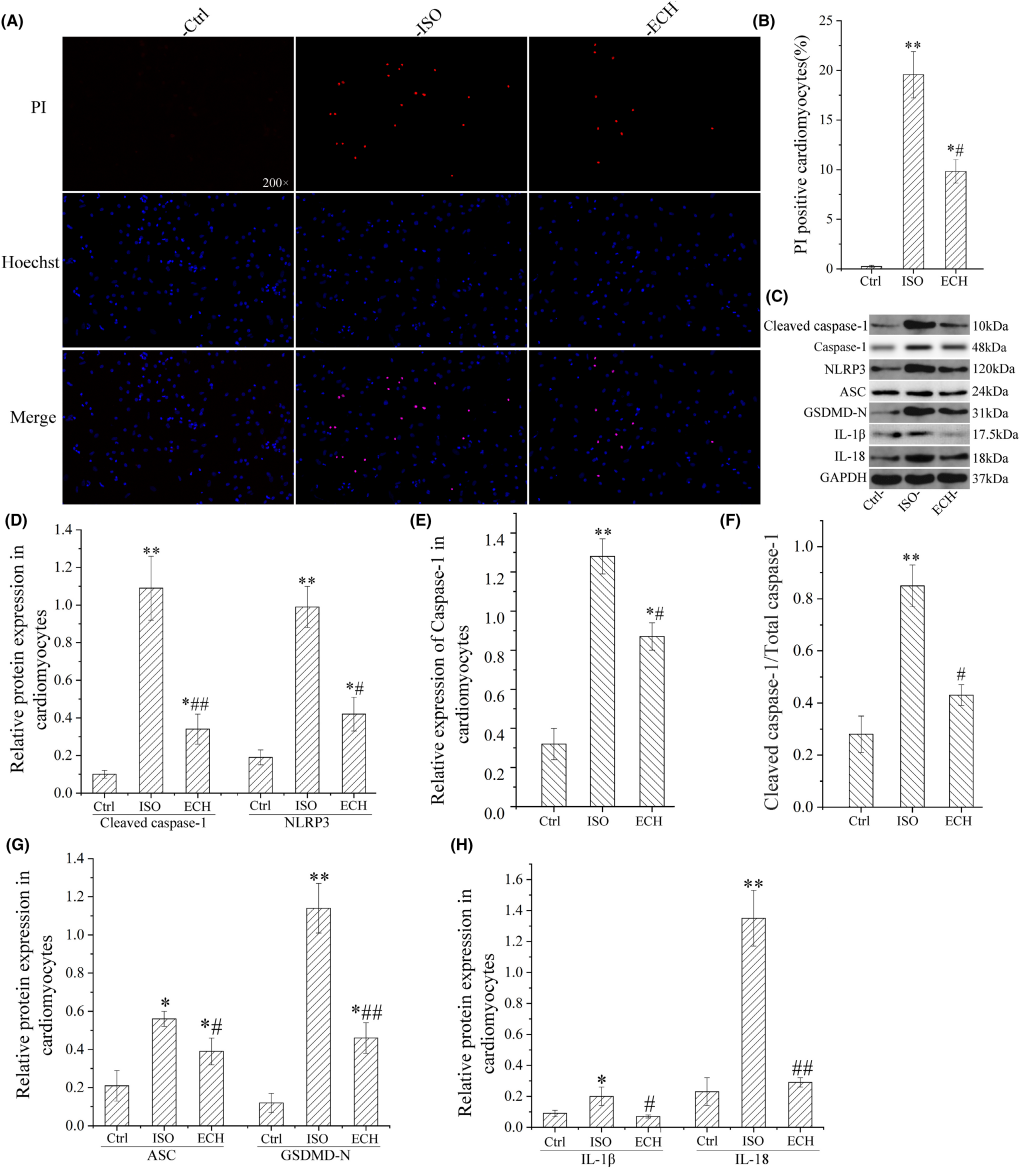
ECH reduced cardiomyocyte pyroptosis in primary cardiomyocytes of neonatal rat induced by ISO. (A) Representative Hoechst/PI staining image of cardiomyocytes treated with Ctrl, ISO, ECH. (B) Statistical histogram of PI‐positive rate of cardiomyocytes. (C) Representative bands of Western blot of cleaved caspase‐1, caspase‐1, NLRP3, ASC, GSDMD‐N, IL‐1β and IL‐18 in cardiomyocytes treated with Ctrl, ISO, ECH. (D–H) Statistical histogram of relative cleaved caspase‐1, caspase‐1, NLRP3, ASC, GSDMD‐N, IL‐1β, IL‐18 expression/GAPDH and the ratio of cleaved caspase‐1/caspase‐1. All *p < 0.05 versus Ctrl, **p < 0.01 versus Ctrl; #*p* < 0.05 versus ISO, ##*p* < 0.01 versus ISO. Error bars represent *SD*.

Primary cardiomyocytes of neonatal rat were used, caspase‐1 mediated pyroptotic rate of cardiomyocytes was measured by flow cytometry, the results indicated that there was a significant increase in pyroptotic rate, and ECH caused an obvious decrease of caspase‐1 mediated pyroptosis. ASC speck formation is an important readout for NLRP3 inflammasome activation; it was also detected by IFC. These ASC specks were around 2 μm and aggerated in the paranuclear area of the cells. The data showed that ASC speckles positive cells were markedly increased in ISO treatment; however, they were significantly reduced by ECH treatment, as shown in Figure 3. The results of Hoechst/PI staining furtherly showed that there were many PI‐positive cell in ISO group, but these cells were reduced in ECH group. That was to say, a large number of pyroptosis of cardiomyocytes were induced by ISO, but in ECH group the pyroptosis quantity was few and ECH effectually reduced cardiomyocytes pyroptosis, as shown in Figure 4A,B. Consistent with the results in vivo, the protein markers of pyroptosis (Cleaved caspase‐1, caspase‐1, the ratio of cleaved caspase‐1/caspase‐1, NLRP3, IL‐1β, IL‐18, GSDMD‐N and ASC) in primary cardiomyocytes of neonatal rat were all distinctly up‐regulated in ISO group, and ECH significantly decreased the expression of these proteins, as shown in Figure 4C–H.

### 3.4 ECH obviously inhibited NOX2, NOX4, p‐SAPK/JNK, ROS and ER stress induced by ISO in vitro

**FIGURE 5:**
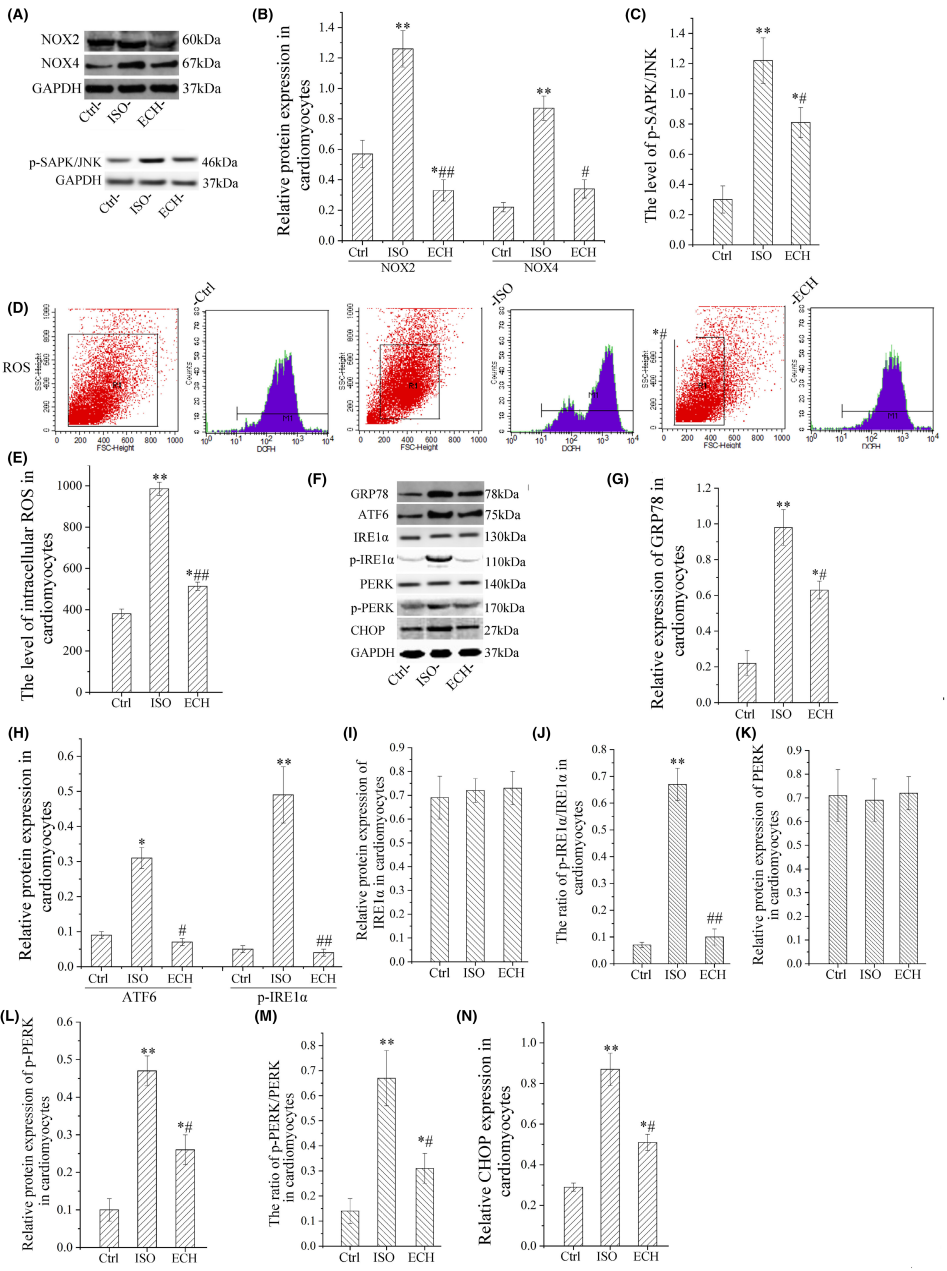
ECH down‐regulated p‐SAPK/JNK, NOX2, NOX4, ROS and ER stress in primary cardiomyocytes of neonatal rat induced by ISO. (A) Representative immunohistochemical image of NOX2, NOX4 and pSAPK/JNK in Ctrl, ISO and ECH‐treated cardiomyocytes. (B, C) Statistical histogram of NOX2, NOX4 and pSAPK/JNK relative expression/GAPDH. (D) Representative flow cytometry image of ROS level. (E) Intracellular ROS level in Ctrl, ISO and ECH‐treated cardiomyocytes. (F) Representative bands of Western blot of GRP78, ATF6, IRE1α, p‐IRE1α, PERK, p‐PERK and CHOP in Ctrl, ISO and ECH‐treated cardiomyocytes. (G–N) Statistical histogram of relative GRP78, ATF6, p‐IRE1α, p‐PERK, CHOP expression/GAPDH and the ratios of pIRE1α/ IRE1α, p‐PERK/PERK. All *p < 0.05 versus Ctrl, **p < 0.01 versus Ctrl; #*p* < 0.05 versus ISO, ##*p* < 0.01 versus ISO. Error bars represent *SD*.

The protein expression of NOX2, NOX4, p‐SAPK/JNK and the intracellular ROS level were all increased in primary cardiomyocytes of neonatal rat treated with ISO, but ECH obviously decreased the levels of NOX2, NOX4 and p‐SAPK/JNK and also reduced the ROS level. The expression of protein markers of ER stress GRP78, p‐IRE1α, p‐PERK, ATF6 and CHOP was all significantly increased, ratios of p‐IRE1α/IRE1α and p‐PERK/PERK were also increased, and these changes were effectively reversed by ECH, but the expression of IRE1α and PERK has no significant difference in among three groups, as shown in Figure 5. To summarize, our results indicated that ECH suppressed cardiomyocyte pyroptosis and protected against HF induced by ISO via inhibiting NADPH/ROS/ER stress.

## 4 DISCUSSION

In recent years, studies have confirmed that pyroptosis promoted cardiac hypertrophy, fibrosis, dysfunction and HF, consistent with this, the present study showed that pyroptosis was closely associated with ISO induced HF, we further indicated that ECH exerted cardioprotective effects by inhibiting cardiomyocytes pyroptosis, and the underlying mechanisms might be ECH down‐regulated NADPH/ROS/ER stress.

Studies have indicated that ECH has potent anti‐oxidation property via reducing intracellular ROS production in other kinds of cells,^22^ but the underlying mechanisms are uncertain. The present study shows that ECH can down‐regulate expression of NOX2 and NOX4 and reduce intracellular ROS in vitro and in vivo, and these results are complementary to its antioxidant pharmacological action in cardiomyocytes of HF.

A recent study shows that ECH can inhibit NLRP3 inflammasome signalling pathway and prevent spinal cord injury,^23^ we further find that ECH inhibits NLRP3 inflammasome associated cardiomyocyte pyroptosis, because ROS act as a major trigger of NLRP3 inflammasome activation,^10^ so, it suggests that ECH inhibit NLRP3 inflammasome signalling pathway and play the anti‐pyroptotic effect via down‐regulating NADPH/ROS. As mentioned above, ROS and ER stress activate and enhance each other as a signal circuit, the present study indicated that ECH effectively suppresses NADPH/ROS and ER stress, it controls the vicious circle and exert the anti‐pyroptotic and cardioprotective effects. Recently, the intercellular communication, ‘cross‐talk’, between ROS derived from NOX and mitochondria, termed ‘ROS‐induced ROS release’, has been proposed as a mechanism for ROS amplification at distinct subcellular compartments^24^; our previous study confirmed that ECH inhibited mitochondria ROS and protected mitochondrial function of cardiomyocytes^18^ via upregulating SIRT1/FOXO3/MnSOD axis; it seems that ECH also has an effect on ‘ROS‐induced ROS release’. So, it indicated that ECH reduces intracellular ROS and exerts the anti‐pyroptotic and cardioprotective effects through multiple mechanisms.

Interestingly, now a recent study indicated that ECH inhibited the phosphorylation levels of Raf/MEK/ERK signalling pathway and subsequently reduced pyroptosis in non‐small cell lung cancer cells.^25^ The inconsistent results may be due to different cell types. For instance, downregulation of the expression of inflammasomes mediating pyroptosis led to the cell proliferation, while downregulation of GSDMD significantly facilitated the cell proliferation of gastric cancer, but in heart, the expression of inflammasomes and GSDMD all elevated and induced cardiomyocytes pyroptosis and subsequently led cardiac hypertrophy, fibrosis, apoptosis, inflammation and dysfunction.^6^, ^26^ So, ECH may work differently in different cells.

ECH is the main extract and active substance of Cistanche, and is a hotspot in the research of Chinese herbal medicine in recent years because of its wide pharmacological functions. Recently, studies find that ECH decreased the phosphorylation levels of I kappa B and NF‐kappa B(p65) in interstitial cystitis,^27^ and the activation of this signalling pathways is enhanced during HF and promoted HF. In addition, ECH elevated FOXO1 expression in the hippocampus, activated Akt/ERK signalling, both the signalling pathways mediated cardioprotection effects.^28^, ^29^ Presumably, ECH protected against HF also via regulating NF‐kappa B, FOXO1 and Akt/ERK signalling pathways. In the present study, we found that ECH down‐regulated phosphorylation levels of SAPK/JNK, the activation of JNK mediated cardiac hypertrophy, apoptosis, interstitial fibrosis, remodelling and dysfunction and inhibited the activation of JNK can protect against cardiac remodelling.^30^, ^31^, ^32^, ^33^, ^34^ So, JNK inhibition was involved in the mechanism of ECH actions.

The results reveal the pharmacological mechanisms of ECH protect against cardiomyocytes pyroptotis and improve heart function of HF, and provide evidences for the development of new drugs. So, our study suggests that ECH is a potential drug for inhibition cardiomyocyte pyroptosis and treatment of HF.

### 4.1 Limitations

There are some limitations in our study. Firstly, the NADPH oxidase activity is an direct indicator of function of NOX2 and NOX4, but we have not measured because of design limitation. Secondly, in vitro experiments we used the primary cardiomyocytes of neonatal rat, there are difference in the activity, biological characteristics and molecular expression between human cardiomyocytes, and this may cause the study results to be inconsistent with it in humans.

## AUTHOR CONTRIBUTIONS

**HongYuan Bai:** Resources (equal); software (equal); supervision (equal); writing – original draft (equal). **Chunhong Luan:** Methodology (equal); software (equal); validation (equal); visualization (equal). **Yixuan Duan:** Conceptualization (equal); data curation (equal); formal analysis (equal); software (equal); validation (equal); visualization (equal). **YaJuan Ni:** Conceptualization (lead); funding acquisition (lead); methodology (lead); resources (lead); writing – original draft (lead). **Jing Zhang:** Conceptualization (equal); data curation (equal); formal analysis (equal); funding acquisition (equal); investigation (equal); software (equal); supervision (equal). **Wenjing Zhu:** Data curation (equal); formal analysis (equal); methodology (lead); project administration (equal); software (supporting); supervision (equal); validation (equal); writing – review and editing (equal).

## FUNDING INFORMATION

This work is supported by Shaanxi Provincial Key Research and Development Projects (2022SF‐293). In addition, we thank for the financial assistance.

## CONFLICT OF INTEREST

The authors declare that they have no competing interests.

## Data Availability

The data used to support the findings of this study are available from the corresponding author upon request.
